# Control Beliefs, Age, and Objectification Experiences in Younger and Older Women

**DOI:** 10.1093/geroni/igab046.212

**Published:** 2021-12-17

**Authors:** Aurora Sherman

**Affiliations:** Oregon State University, Corvallis, Oregon, United States

## Abstract

Control beliefs show age-related patterns; mastery decreases in adulthood, while constraints beliefs often increase. However, there is a great deal of individual variation. This paper addresses antecedents of control beliefs, with attention to experiences and beliefs related to sexual objectification, which have particular impact for women. In this study, younger women (N = 132, M = 20.93) and older women (N = 86, M = 67.83) were surveyed regarding their experiences with sexual objectification and constraints beliefs. Multiple regression analyses revealed higher self-objectification was associated with higher constraints (R2 = .09**) and lower mastery (R2 = .11**) but reports of body evaluation and sexual advances were not associated with control beliefs. Further, there were no interactions of either objectification scale with age. These results suggest that objectification may be an important part of the aging experience across the life course, not just in young adulthood.

